# An Alternative Pin1 Binding and Isomerization Site in the N-Terminus Domain of PSD-95

**DOI:** 10.3389/fnmol.2020.00031

**Published:** 2020-03-18

**Authors:** Jary Y. Delgado

**Affiliations:** Department of Neurobiology, University of Chicago, Chicago, IL, United States

**Keywords:** postsynaptic density protein 95, proline-directed phosphorylation, excitatory synaptic transmission, Pin1, *cis-trans* isomerization

## Abstract

Phosphorylation-dependent peptidyl-prolyl *cis-trans* isomerization plays key roles in cell cycle progression, the pathogenesis of cancer, and age-related neurodegeneration. Most of our knowledge about the role of phosphorylation-dependent peptidyl-prolyl *cis-trans* isomerization and the enzyme catalyzing this reaction, the peptidyl-prolyl isomerase (Pin1), is largely limited to proteins not present in neurons. Only a handful of examples have shown that phosphorylation-dependent peptidyl-prolyl *cis-trans* isomerization, Pin1 binding, or Pin1-mediated peptidyl-prolyl *cis-trans* isomerization regulate proteins present at excitatory synapses. In this work, I confirm previous findings showing that Pin1 binds postsynaptic density protein-95 (PSD-95) and identify an alternative binding site in the phosphorylated N-terminus of the PSD-95. Pin1 associates *via* its WW domain with phosphorylated threonine (T19) and serine (S25) in the N-terminus domain of PSD-95 and this association alters the local conformation of PSD-95. Most importantly, I show that proline-directed phosphorylation of the N-terminus domain of PSD-95 alters the local conformation of this region. Therefore, proline-directed phosphorylation of the N-terminus of PSD-95, Pin1 association, and peptidyl-prolyl *cis-trans* isomerization may all play a role in excitatory synaptic function and synapse development.

## Introduction

The postsynaptic density (PSD) of excitatory synapses is a highly crowded space composed of transsynaptic proteins, extracellular matrix constituents, surface receptors, ion channels, and scaffolding proteins. The scaffolding proteins at the PSD are essential elements required for the enrichment of ionotropic α-amino-3-hydroxy-5-methyl-4-isoxazolepropionic acid (AMPA)-type and N-methyl-D-aspartate receptor (NMDAR)-type glutamate receptors at the PSD (Sheng and Hoogenraad, 2007). At a given PSD, there are over 300 copies of the postsynaptic density protein-95 (PSD-95).

Although PSD-95 is a rather stable element of the PSD, capable of sustaining harsh biochemical extractions, PSD-95 synaptic stability is regulated following the induction of synaptic plasticity (Migaud et al., 1998; Colledge et al., 2003; Sun and Turrigiano, 2011). In particular, the induction of synaptic plasticity, namely NMDAR-dependent long-term depression (LTD), increases the phosphorylation state of the N-terminus domain of PSD-95 and this event regulates its stability, clustering, proteolytic cleavage, and PSD-95 palmitoylation (Colledge et al., 2003; Morabito et al., 2004; Xu et al., 2008; Nelson et al., 2013; Zhang et al., 2014; Chowdhury et al., 2018). Specifically, the induction of NMDAR-dependent LTD increases threonine 19 (T19) and serine (S25) phosphorylation of PSD-95 (Nelson et al., 2013). Despite significant advances on this topic, there is a lack information about potential binding partners interacting with phosphorylated T19 and S25 in PSD-95 that may regulate the stability following its increase in phosphorylation.

A potential protein that could interact with phosphorylated T19 and S25 is the phosphorylation-specific peptidyl-prolyl *cis-trans* isomerase (Pin1) as Pin1 has been shown to bind phosphorylate T287, S290, and T295 (Antonelli et al., 2016). Pin1 is a small cytosolic enzyme that exclusively binds phosphorylated S/T-Proline sites. Pin1 has two major domains: an N-terminal WW domain and a C-terminal peptidyl-prolyl isomerase (PPIase) domain. The WW domain of Pin1 recruits the protein to the phosphorylated serine/threonine-proline (S/T-P) residues of its target protein and the catalytically active peptidyl-prolyl isomerase (PPIase) triggers the *cis-trans* peptidyl-prolyl isomerization (Yaffe et al., 1997; Verdecia et al., 2000; Lu et al., 2007).

Pin1 serves various functions in neuronal and excitatory synaptic physiology. In the hippocampus, Pin1 is involved in late-phase LTP *via* the regulation of dendritic protein synthesis (Westmark et al., 2010). At excitatory synapses of striatal MSN and pyramidal neurons of the hippocampus, Pin1 regulates NMDAR currents by interacting with the phosphorylated T287, S290, and T295 in PSD-95 (Park et al., 2013; Antonelli et al., 2016); however, there is reason to believe that Pin1 binds additional sites in PSD-95. A closer inspection of the data presented in Antonelli et al. (2016) shows that binding is still observed in the deletion mutant of PSD-95 (Δ287–295, Figure 2B, Antonelli et al., 2016), which is the presumed binding region in PSD-95. Furthermore, the phosphomutant of PSD-95 where T287, S290, and T295 are replaced to alanine show increased cellular proteolysis. The proteolytic fragments of PSD-95 do not contain the N-terminus phosphorylation sites T19 and S25 which can serve as alternative Pin1 binding sites (Xu et al., 2008). Therefore, the observed loss in Pin1 binding to the PSD-95 alanine mutants could be due to the loss of an alternative binding site at the N-terminus domain of PSD-95. Therefore, the question of whether Pin1 associate with the phosphorylated serine-threonine residues in the N-terminus domain of PSD-95 has not been thoroughly examined yet.

**Figure 1:**
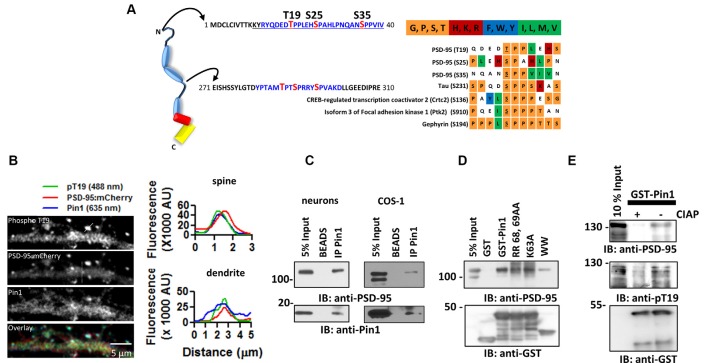
The peptidylprolyl *cis-trans* isomerase NIMA-interacting 1 (Pin1) interacts with postsynaptic density protein-95 (PSD-95) *via* its WW domain. **(A)** (Left) Schematic diagram of PSD-95 indicating putative Pin1 binding sites (shown in blue). (Right) Amino acid sequence alignment oriented around the S/T-P motifs. A chart was generated using the PRALINE algorithm. Color-code based on amino acid groups; shown above in abbreviated form. **(B)** Pin1 co-localizes with phosphorylated PSD-95 *in vivo*. Representative confocal images of hippocampal neurons transfected with PSD-95::mCherry (2^nd^ image) and immunostained against phospho-T19 (Top) and Pin1 (3^rd^ image) and overlay image (lower). Line scans above a dendrite and a dendritic spine (white lines) showing good co-localization on spines (top) and dendrites (bottom), right graphs. **(C)** PSD-95 Pin1 interaction is present* in vivo*. Neurons experiments performed on endogenous proteins. For COS-1 lysates were transfected with full-length PSD-95::EGFP and Pin1 and immunoprecipitated with the anti-Pin1 antibody. Complexes were subjected to Western immunoblotting with anti-PSD-95 and anti-Pin1 antibodies, *n* = 3. **(D)** Pin1 WW domain is sufficient for PSD-95 interaction. COS cells homogenate expressing PSD-95::EGFP were incubated with GST, GST-Pin1, GST-Pin RR 68, 69 AA, GST-Pin K63A or GST-Pin1 WW domain. Complexes were subjected to Western immunoblotting with anti-PSD-95 and GST antibodies, *n* = 4. **(E)** PSD-95 Pin1 interaction is regulated by phosphorylation. COS cell homogenate expressing PSD-95::EGFP were incubated with (−) or without (+) CIAP followed by incubation with GST-Pin1. Complexes were subjected to Western immunoblotting with anti-PSD-95, anti-pT19 and GST antibodies.

**Figure 2:**
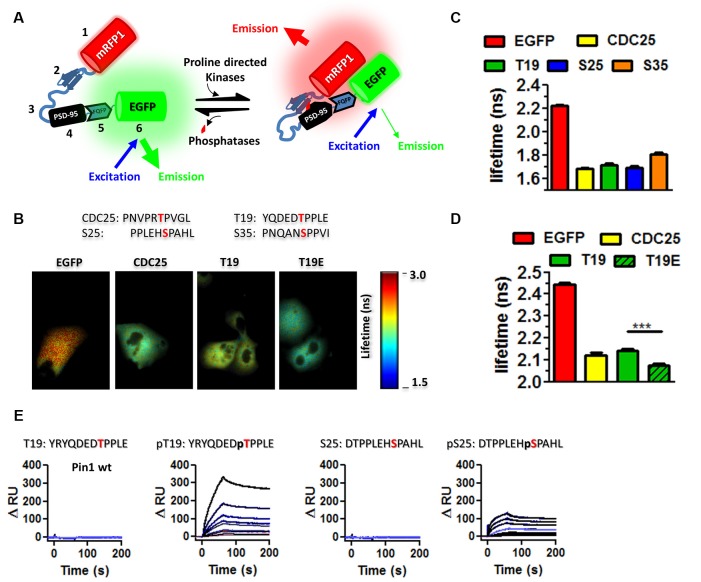
Pin1 WW domain binds the N-terminus domain of PSD-95. **(A)** EKAR constructs used to screen for interactions with phospho-sequences in PSD-95. EKAR components: 1. mRFP1, 2. Pin1-WW domain, 3. glycine linker, 4. CDC25c substrate peptide or PSD-95 phospho-peptides, 5. ERK docking domain, 6. mEGFP, red diamond represent the addition of phosphate group. **(B)** EGFP fluorescence lifetime images of COS cells transfected with EGFP, EKAR_cyto_, EKAR_cyto_T19, EKAR_cytoT19E_. The substrate peptide sequence is shown above. **(C)** The WW domain binds to T19, S25, and S35. Summary plot showing the average EGFP fluorescence lifetime from fixed mounted COS cells EGFP 2.225 ± 0.078 *n* = 575; CDC25 1.679 ± 0.2001 *n* = 256; T19 1.715 ± 0.1753; S25 1.689 ± 0.196 ns *n* = 200; S35 1.808 ± 0.0139. Graphs show the mean ± SEM. **(D)** Summary plot showing the average EGFP fluorescence lifetime from live COS cell imaging showing equal affinity to CDC25 peptide EGFP 2.444 ± 0.0072 *n* = 136; CDC25 2.122 ± 0.12 *n* = 142; T19 2.143 ± 0.082; T19E 2.074 ± 0.078 ns *n* = 87. A Kruskal–Wallis test with Dunn’s multiple comparison test ***p < 0.0001. **(E)** Kinetic interaction of GST-Pin1 with phospho and non-phosphorylated peptides in the N-terminus domain of PSD-95 as visualized by SPR in a Biacore 3000 apparatus for wt GST-Pin1. The association and dissociation phases were monitored for 200 s by following the change in RU for different concentrations of GST fusion proteins in μM: 0.25, 0.125, 0.063, 0.05, 0.0313, 0.025, 0.0156, 0.01, 0.0078 for phosphorylated or non-phosphorylated peptides. The peptide sequence is shown on top.

In this work, I demonstrate that Pin1 binds phosphorylated T19 and S25 in the N-terminus domain of PSD-95. Phosphorylation of these residues triggers a change in PSD-95 conformation and Pin1 association with the phosphorylated residues restores the conformation of this domain. These findings position Pin1 as a key protein with the potential to regulate forms of synaptic plasticity dependent on phosphorylation of the N-terminus domain of PSD-95.

## Materials and Methods

### Cloning and cDNA Plasmids

The plasmid encoding PSD-95::EGFP was a gift from S. Okabe (Tokyo University, Japan). The triple T19A, S25A and S35A (N3A-PSD-95) and the double T287A and S295A (**C2A**-PSD-95) PSD-95::EGFP mutants were generated using site-directed mutagenesis following the manufacturer’s recommendations (Agilent Technologies, Santa Clara, CA, USA) and sequence verified. For the N3A-PSD-95 mutant, we first introduced the T19A and S25A double mutation using the following primer set (Table). We then introduced the S35A mutation. For the C2A mutant, we introduced the T287A and S295A double mutation. The GST-Pin1 K63A and GST-Pin1 WW (GST-Pin1) were obtained from Addgene, plasmid ID# 19027 as described in Yaffe et al. (1997) mutant was generated using the following primer set (Table). The T19, T19E, S25, and S35 PSD-95 peptide sequences were cloned into the EKAR construct using the following primer set (Table).

**Table:** 

Mutant	Sense	Antisense
PSD-95 T19A and S25A	GAAATACCGCTACCAAGATGAAGACGCGCCCCCTCTGGAACACGCGCCGGCCCACCTCCCCAACCAGGCCAATTC	GAATTGGCCTGGTTGGGGAGGTGGGCCGGCGCGTGTTCCAGAGGGGGCGCGTCTTCATCTTGGTAGCGGTATTTC
PSD-95 S35A	GGCCCACCTCCCCAACCAGGCCAATGCGCCCCCTGTGATTGTCAACACGGACAC	GTGTCCGTGTTGACAATCACAGGGGGCGCATTGGCCTGGTTGGGGAGGTGGGCC.
PSD-95 T287A and S295A	GGGCACTGACTACCCCACAGCCATGGCGCCCACTTCCCCTCGGCGCTACGCGCCTGTGGCCAAGGACCTGCTGGGGG	CCCCCAGCAGGTCCTTGGCCACAGGCGCGTAGCGCCGAGGGGAAGTGGGCGCCATGGCTGTGGGGTAGTCAGTGCCC.
GST-Pin1 K63A	CGCACCTGCTGGTGGCGCACAGCCAGTCAC	TGACTGGCTGTGCGCCACCAGCAGGTGCG
GST-Pin1 WW	GCCCAGCGGCAACAGCAGCAGTGGTGGCTAAAACGGGCAGGGGGAGCCTGCCAGGG	CCCTGGCAGGCTCCCCCTGCCCGTTTTAGCCACCACTGCTGCTGTTGCCGCTGGGC
GST-Pin1 R68A, R69A	GCACCTGCTGGTGAAGCACAGCCAGTCAGCGGCGCCCTCGTCCTGGCGGCAGGAGAAG	CTTCTCCTGCCGCCAGGACGAGGGCGCCGCTGACTGGCTGTGCTTCACCAGCAGGTGC
EKAR T19	GTGGTCGACGGTACCGCGGACCGGTTACCAAGATGAAGACACGCCCCCTCTGGAACACGCAAAGCTGTCATTCCAATTCCCGC	GCGGGAATTGGAATGACAGCTTTGCGTGTTCCAGAGGGGGCGTGTCTTCATCTTGGTAACCGGTCCGCGGTACCGTCGACCAC
EKAR T19E	GTGGTCGACGGTACCGCGGACCGGTTACCAAGATGAAGACGAGCCCCCTCTGGAACACGCAAAGCTGTCATTCCAATTCCCGC	GCGGGAATTGGAATGACAGCTTTGCGTGTTCCAGAGGGGGCTCGTCTTCATCTTGGTAACCGGTCCGCGGTACCGTCGACCAC
EKAR S25	GTGGTCGACGGTACCGCGGACCGGTCCCCCTCTGGAACACAGCCCGGCCCACCTCCCCGCAAAGCTGTCATTCCAATTCCCGC	GCGGGAATTGGAATGACAGCTTTGCGGGGAGGTGGGCCGGGCTGTGTTCCAGAGGGGGACCGGTCCGCGGTACCGTCGACCAC
EKAR S35	GTGGTCGACGGTACCGCGGACCGGTCCCAACCAGGCCAATTCTCCCCCTGTGATTGTCGCAAAGCTGTCATTCCAATTCCCGC	GCGGGAATTGGAATGACAGCTTTGCGACAATCACAGGGGGAGAATTGGCCTGGTTGGGACCGGTCCGCGGTACCGTCGACCAC

### Expression and Purification of the GST Fusion Proteins

Cell amplification, protein harvesting, and isolation proceeded as described in Sainlos et al. (2009) with the exception that purified proteins were dialyzed, flash-frozen and stored at −80°C in 20 mM Tris-HCl buffer (pH 7.5) containing 0.1 M NaCl, 5 mM DTT and 20% Glycerol.

### GST-Pulldown

COS-7 or COS-1 cells were transfected with PSD-95::EGFP using X-TremeGENE (Roche, Basel, Switzerland). Two days post-transfection cells were scraped off and lysed in buffer (in mM), 50 Tris-HCl, 200 NaCl, 100 NaF, 10% Glycerol, 1% Triton X-100, pH 8) containing protease inhibitor cocktail III (Calbiochem) and homogenized using three brief (1–3 s long) pulses on an ultrasonic homogenizer. Cells were spun at 5,000 rpm for 5 min and the supernatant was collected. Protein concentration was measured using the BCA method (Pierce). Between two hundred micrograms of total protein (depending on cell confluence) were incubated with 200 μg of GST fusion proteins and incubated for a minimum of 4 h followed by 2 h incubation with 20 μl of glutathione coated magnetic beads pre-blocked with 1% BSA (Pierce). Protein complexes were thoroughly washed with 200 μl and eluted in 50 μl of homogenization buffer containing 10 mM reduced glutathione pH ~8.0.

### Pin1 Isomerase Spectrophotometer Assay

The peptide substrate N-Succinyl-Ala-Glu-Pro-Phe-p-Nitroanilide (Peptides International, Inc., Louisville, KY, USA) was dissolved to 16 mM in DMSO. One microliter of the peptide stock solution was diluted in 100 μl of 480 mM LiCl in trifluoroethanol for 10 min before the start of the reaction. The reaction was carried out in cold buffer (0.1 M NaCl, 50 mM Hepes, 2 mM DTT and 0.04 mg/ml BSA, pH 7.0) containing 1–2 μg of GST-Pin1, 6 μg of α-chymotrypsin (Sigma–Aldrich, St. Louis, MO, USA) in 1 mM HCl and 0.5 μg of the peptide substrate. The absorbance change was immediately measured at 390 nm using a UV-Vis spectrophotometer (UV-1800, Shimadzu) at room temperature in cold buffer.

### Isomerization of Full-Length PSD-95

This assay was adapted (Stukenberg and Kirschner, 2001). Transfected COS-7 cells were homogenized as described previously without serine protease inhibitors. 30 μg of total protein were aliquoted into individual Eppendorf tubes (4°C) labeled: input, BSA or Pin1. Input samples were quickly denatured by the addition of 6× Laemmli sample buffer (LB) and boiled at 85°C for 10 min. BSA or Pin1 samples received 1 μl of α-chymotrypsin at 200 ng/μl in 0.08 M Tris HCl buffer, pH 7.8 containing 0.1 M calcium and 1 μg of BSA or Pin1. The reactions were quickly mixed and left to proceed undisturbed for 30 s at 25°C, stopped with the addition of 6× LB, and boiled for 10 min.

### Immunoprecipitation

COS-7 cells were transfected with PSD-95::EGFP and Pin1. Whole-cell lysates were incubated with 4 μg of the anti-Pin1 antibody for 4 h followed by a 2 h incubation with 30 μl of Sepharose A coated beads pre-blocked for 1 h. Immunoprecipitated protein complexes were thoroughly washed 3X with 200 μl of homogenization buffer. Protein complexes were eluted in 50 μl of lysis buffer with 2× LB. For immunoprecipitation of PSD-95 from cultured neurons, a 20 mm dish at a confluency of 200,000 cells was scraped in 100 μl of HB containing 50 Tris-HCl, 200 NaCl, 100 NaF, 10% Glycerol, 0.5% deoxycholate, pH 8 containing protease inhibitor cocktail III, briefly sonicated and diluted in equal volumes of 50 Tris-HCl, 200 NaCl, 100 NaF, 10% Glycerol, 1% Triton X-100, pH 8 containing protease inhibitor cocktail III.

### Western Blotting

**Figure 3:**
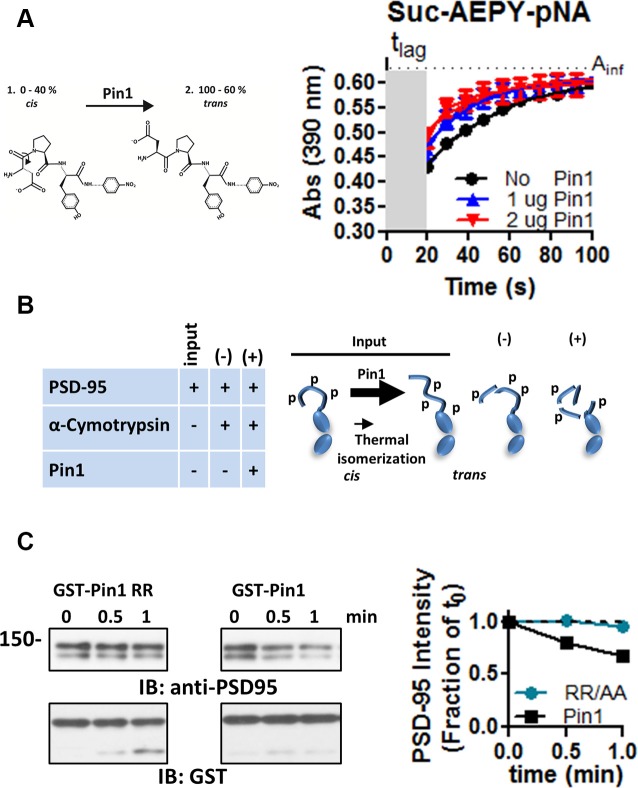
Pin1 isomerizes full-length PSD-95. **(A)** A *cis-trans* isomerization scheme mediated by Pin1 on the succinyl-AEPY-p-nitronilide peptide. For representation, only EPY-p-nitronilide is shown. The predicted amounts of each isomer in solution (see “Materials and Methods/References” secton). The cis-trans isomerization assay to test the functionality of the purified GST-Pin1. **(B)** (Left) Table showing the experiment design for the α-chymotrypsin-coupled *cis-trans* isomerization assay. (Right) Scheme of the Pin1-mediated isomerization reaction, only N-terminus, PDZ1 and PDZ2 are shown. Input condition contains both *cis* and *trans*-N-terminus PSD-95, (−) PSD-95 product in the presence of α-chymotrypsin alone and (+) PSD-95 product in the presence of Pin1 with α-chymotrypsin. **(C)** Immunoblot showing the results of the *in vitro* α-chymotrypsin *cis-trans* isomerization of full-length PSD-95. Homogenates of COS cells expressing full-length PSD-95::EGFP were incubated with the isomerase dead GST-Pin RR68, 69AA or GST-Pin1 and treated with 0.1 μg of chymotrypsin. Reactions were subjected to Western immunoblotting with anti-PSD-95 and GST antibodies. (Middle) Quantification of immunoblot band intensities normalized to time 0 for the 135 kDa band (*n* = 1).

**Figure 4:**
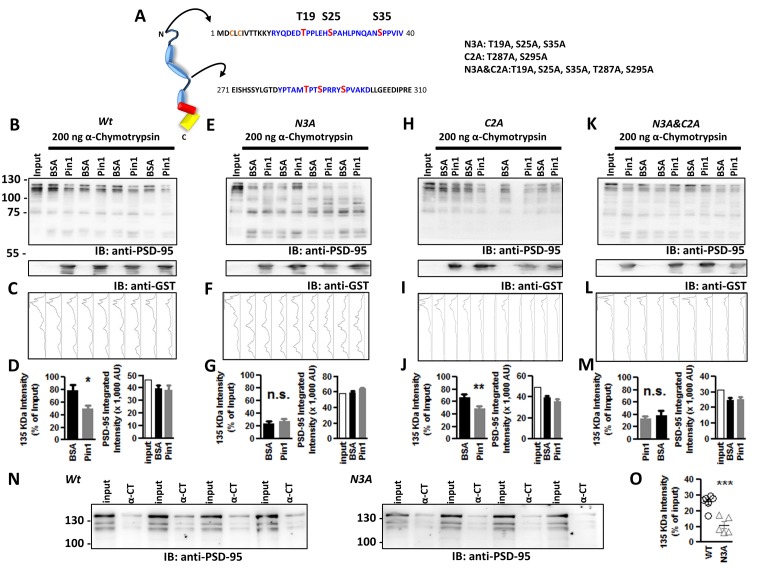
Phospho-T19, S25, and S35 in the N-terminus domain of PSD-95 are sites of Pin1 isomerization. **(A)** Schematic diagram of PSD-95 indicating putative Pin1 binding sites (shown in blue) and phosphorylated sites in red. Abbreviated mutant is shown on right including the sites mutated on each one. **(B)** Immunoblot showing the result of the *in vitro* a-chymotrypsin cis-trans isomerization of wt PSD-95, **(E)** the N3A, **(H)** the C2A, and **(K)** the N3A/C2A mutant. COS cells homogenates expressing wt or mutants of PSD-95::EGFP were incubated with 1 mg BSA or GST-Pin1 and subjected to Western immunoblotting with anti-PSD-95 and anti-GST antibodies. **(C,F,I,L)** Integrated band intensity for all the bands. Each lane corresponds to the PSD-95 immunostained column above. **(D,G,J,M)** Quantification of immunoblot intensities normalized to input lane value for the 135 kDa band. **(D)** Wt, BSA 78.78 ± 8.628% vs. Pin1 49.74 ± 5.722%; *n* = 9, *p < 0.05 unpaired t-test. **(G)** N3A, T19A, S25A, S35A PSD-95::EGFP triple mutant BSA 23.78 ± 3.246% vs. Pin1 27.44 ± 3.689%, *n* = 8; **(J)** C2A T287A, S295A PSD-95::EGFP double mutant BSA 67.38 ± 13.39% vs. Pin1 49.68 ± 10.10%, *n* = 8 and **(M)** the N3A&C2A T19A, S25A, S35, T287A, S295A PSD-95::EGFP Penta mutant BSA 34.47 ± 7.712% vs. Pin1 37.69 ± 3.426%, *n* = 6. *p < 0.05 and **p < 0.01 unpaired *t*-tests. For all (right bar graph) the difference in rates of PSD-95 degradation by Pin1 is not explained by differences in loaded protein. **(N)** Isomerization assay performed as before without the addition of GST-Pin1 to lysates. Cells transfected with wild type PSD-95 or the N3A. **(O)** Quantification of immunoblot band intensities normalized to input lane values for the 135 KDa band 25.72 ± 1.87%, *n* = 6 for PSD-95::EGFP and 10.99 ± 2.37%, *n* = 5 for PSD-95::EGFP N3A, ***p < 0.0008. Four independent pairs of reactions shown. All values are reported as mean ± SEM. n.s., not significant.

Proteins were subjected to SDS-PAGE electrophoresis, transferred onto PVDF membranes and immunoblotted using the anti-PSD-95 antibody and anti-GST. The Pierce West Femto ECL substrate was used to reveal the immune complex. Images were taken using a Syngene apparatus (Figures 3, 4) or on X-Ray film (Figure 1) and analyzed using ImageJ. We only analyzed images obtained on the Syngene apparatus. Brightness and contrast are adjusted.

### EGFP Fluorescence Lifetime Imaging

Live-cell imaging for Figure 1C and fixed/mounted cell imaging for Figure 1D. Cells were imaged in extracellular solution, or mounted in Vectashield, containing (in mM, 125 NaCl, 3 KCl, 10 HEPES, 10 Glucose, 2 CaCl and 1 MgCl pH 7.34) using a 40× 1.25 NA HCX PL Apo oil immersion objective in a modulated Light-Emitted-Diode 451 nm (3W). Lifetime acquisition and measurements were performed on an inverted Leica DMI6000B (Leica Microsystem, Wetzlar, Germany) microscope equipped with a LIFA frequency-domain lifetime attachment (Lambert Instruments, Roden, The Netherlands) and the manufacturer’s LI-FLIM software. Lifetimes were referenced with a 1 mM solution of fluorescein in phosphate-buffered saline (PBS; pH 10), 4.00 ns lifetime. Measurements were obtained offline from an area encompassing most of the cell excluding the nucleus as in Zhang et al. (2013).

### Hippocampal Cultured Neurons

Preparation of cultured neurons was performed by plating neurons at a density of 100–200K/well of a 6-well plate. In brief, hippocampal neurons from E18 embryos of either sex were cultured on glass coverslips coated with PEI. Neurons were plated in Neurobasal supplemented with B27, 5% FBS and glutamine. Two days post-plating neurons were treated with 1 μM Ara-C to stop glia and microglial proliferation. Feedings were done every 4 days using low cysteine-containing media (Hogins et al., 2011). At day *in vitro* 8–10 neurons were transfected using Lipofectamine 2000 following the manufacturer’s recommendation. Experiments were performed on neurons were between 11 and 20 DIV.

### Immunostaining

Three to five days post-transfection cells were fixed in 4% Paraformaldehyde at room temperature for 20 min. Cells were then rinsed three times with 1× PBS, then 5 min in 50 mM NH4Cl, and three more quick rinses in 1× PBS. Cells were permeabilized in 0.1% Tx100 PBS for 5 min followed by three quick PBS rinses and incubated in 2 mL of 1%BSA in PBS for 45 min followed by incubation in 100 μl of anti-Pin1 (Santa Cruz Biotechnology, Dallas, TX, USA) and the anti-phospho T19 (1:500) for 1 h. Cells were rinsed 3× in PBS and the Alexa fluor 647 anti-mouse (1:500), and Alexa fluor 488 anti-rabbit (1:500) for 1 h in 1% BSA in PBS. Cells were rinsed 5× in PBS, postfix in 4% PFA and mounted in slow fade mounting media (Life Technologies, Carlsbad, CA, USA).

### Spinning Disk Confocal

Cells were imaged using 3-I Marianas live-cell dual-camera Yokogawa CSU-X spinning disk confocal. AxioObserver platform with DualCam and two Evolve EM-CCD cameras, CFP/YFP and R/G cubes using 100× /1.45 oil objective. We used the solid-state 488, 561, and 640 lasers with fiber switcher to excite the corresponding fluorophores as needed. The objective was mounted onto a piezo MadCityLabs piezo Z insert which was used to collect Z-stacks.

### Peptide Synthesis

Peptides were synthesized using 9-fluorenyl methoxycarbonyl (Fmoc) solid-phase peptide synthesis with rink amide 4-methylbenzhydrylamine resin (EMD Millipore). The synthesis was performed on a CEM Liberty automated microwave peptide synthesizer. After removal of Fmoc groups with 30% 4-methylpiperidine and 0.1 M hydroxybenzotriazole (HOBt) in *N*,*N*-dimethylformamide (DMF) at 75°C for 3–4 min, each amino acid or biotin (4 equiv.) was coupled at 75°C for 5–10 min using 4 equiv. of *O*-benzotriazole-*N*,*N*,*N*′,*N*′-tetramethyluronium hexafluorophosphate (HBTU), and 8 equiv. of *N*, *N*-diisopropylethylamine (DIEA). After synthesis, peptides were cleaved from the resin with a 95:2.5:2.5 trifluoroacetic acid (TFA)/triisopropylsilane (TIPS)/water mixture for 3–4 h. Rotary evaporation and precipitation in cold diethyl ether yielded the crude peptide mixture. Crude peptides were purified by HPLC on a C18 Phenomenex Jupiter or Gemini column in a water-acetonitrile gradient containing 0.1% *v*/*v* TFA or NH4OH respectively. Pure fractions were collected and identified using ESI-MS. The combined fractions were subjected to rotary evaporation to remove volatile solvents, frozen, and lyophilized to dryness. The purity of the final lyophilized solid was verified by LCMS.

### Surface Plasmon Resonance

SPR was used to measure the yes or no interaction between GST::Pin1 (and its mutants) and the different PSD-95 peptides using a BIAcore 3000 biosensor. Various biotinylated PSD-95 peptides were bound to the streptavidin matrix of a sensor chip. The immobilization process was carried out at a flow rate of 10 μl/min. Running buffer was used to prepare the control surface. The running buffer consisted of (in mM): 10 HEPES, 150 NaCl, 0.050 EDTA pH 7.4, 0.005% Tween 20. GST:Pin1 and its mutants (analyte) at various concentrations (see “Results” section) were injected with a flow of 10 μl/min over the immobilized peptides. Care was taken to use a low amount of protein to keep the signal below 400 Refractive Units (RU) units. The binding was assessed by monitoring the change in the refractive index (given in arbitrary response units, RU). The association/dissociation phases were monitored for 300 s. After each binding experiment, the sensor chip was regenerated by sequential washing with 0.85% phosphoric acid buffer (Bio-Rad). Several rounds of injections and regeneration were performed.

### Experimental Design and Statistical Analysis

At least two coverslips were used on each data set. Data collection was interleaved, control for time and order effects. Samples from all groups were acquired on a weekly basis to reduce variability, else no data were included in the final analysis. We tested for outliers on a weekly basis and they were eliminated after testing all groups using Prism online calculator at a significance level of *p* < 0.05. Normality testing was performed on every group using D’Agostino and Pearson omnibus normality test. Between-group statistical significance was calculated accordingly for each distribution and experiment design. Data were normalized on a weekly basis to compensate for week to week variability. Numerical averages are presented as mean ± SEM or as box plots Statistical analyses were created using GraphPad Prism 5.0. Exact *p*-values are reported when provided.

**Table:** Antibody table.

Antibody	Company	Catalog #	Concentration	Application
anti-Pin1	Santa Cruz	SC-5340	1\500	Immunofluorescence
			4 μG	Immunoprecipitation
anti-PSD-95	Neuromab	K28/43	1\3,000	Western blot
anti-PT19	Abcam	ab16496	1\500	Immunofluorescence
anti-GST	Thermoscientific	MA4–004	1\5,000	Western blot

## Results

### Pin1 Binds Phosphorylated Threonine 19 in PSD-95 *via* Its WW Domain

PSD-95 contains six phosphorylatable serine/threonine-proline sites, three of these sites—threonine 19 (T19), serine 25 (S25), and serine 35 (S35)—are in the N-terminus domain, and the other three—threonine 287 (T287), serine 290 (S290), and serine 295 (S295)—are within the flexible linker region of PSD-95 between PDZ2 and PDZ3 (Figure 1A; Coba et al., 2009). To evaluate the hypothesis that Pin1 could bind phospho T19, S25 and S35 PSD-95, I performed a sequence alignment between these sites in PSD-95 and other Pin1 binders. The sequence alignment showed that T19, S25, and S35 contain many of the amino acids found in most Pin1 binding partners (Figure 1A right). The T19/S25 phosphorylated form of PSD-95 enriches, biochemically, to the PSD fraction II, but lower amounts can be detected in the PSD fraction number III (Morabito et al., 2004). To test if this interaction occurs* in vivo*, I immunostained cultured neurons with antibodies against Pin1 and phospho-T19. Pin1 showed ubiquitous expression and co-localized with phospho-T19 PSD-95 on dendritic spines (Figure 1B), suggesting that Pin1 is in close proximity to phosphorylated T19-PSD-95 and could interact *in vivo*. The interaction between PSD-95::EGFP and Pin1 were re-examined *via* co-immunoprecipitation and GST pulldown experiments. In both COS-1 and neuronal lysates, Pin1 and PSD-95 co-immunoprecipitated (Figure 1C) and no binding was detected towards the lower molecular weight bands of PSD-95. As stated earlier, the lower molecular fragments of PSD-95 do not contain the N-terminus phosphorylation sites T19 and S25; suggesting that Pin1 interaction with PSD-95 requires the integrity of this domain (Xu et al., 2008).

To determine which Pin1 domain mediates the interaction with PSD-95, I performed a series of GST pull-down assays. GST-Pin1 but not with GST pulled-down PSD-95 (Figure 1D). This interaction was not affected by two isomerase dead mutants [the K63A and R68A, R69A (RR/AA)], and it was also present in beads coated with the WW domain of Pin1. Once again, Pin1 interacted only with full-length PSD-95.

The phosphorylation-dependent binding of Pin1 to PSD-95 was confirmed using the GST pull-down assay with lysates treated with calf intestinal alkaline phosphatase (a broad-spectrum phosphatase). CIAP reduced Pin1 binding to PSD-95::EGFP (Figure 1E).

To evaluate if the phospho-sites in PSD-95 and Pin1 interact in live cells, I performed fluorescence resonance energy transfer/fluorescent lifetime imaging (FRET/FLIM) experiments using the EKAR construct (Harvey et al., 2008). The original EKAR construct contains the Pin1-WW domain fused in frame with a flexible serine/glycine linker and the phosphopeptide of CDC25C. The CDC25 phosphorylatable sequence served as a positive control. In addition, the EKAR construct contains mRFP and EGFP, which, respectively, serve as the acceptor and donor fluorescent proteins (Figure 2A, scheme). In the experimental conditions, the sequence of CDC25C was replaced with a 10-mer phosphorylatable peptide of T19 and S25. The association between the WW domain of Pin1 and the PSD-95 peptides was measured *via* FLIM as a reduction in EGFP lifetime (Figure 2A, scheme right hand). Kinase activity was triggered by the presence of serum in the growing medium. Cells expressing the T19 peptide of PSD-95 showed similar values of EGFP lifetime as cells expressing the CDC25C phosphopeptide (Lu and Zhou, 2007), suggesting that the Pin1 WW domain binds equally well to T19 and T48 in CDC25C (Figures 2B,C). To test if the WW domain prefers T19, S25 or S35 in PSD-95, I repeated the FLIM experiments and noticed similar binding for all peptides, although binding was a little weaker for S35 (Figure 2C, imaging performed in fixed mounted cells). The phosphomimetic forms of the T19 peptide showed increased binding (imaging performed in living cells, Figure 2D right, one-way ANOVA with Bonferroni posttest, *F*_(3,448)_ = 381.3, ***p < 0.001), indicating that Pin1 prefers acidic residues.

Next, surface plasmon resonance (SPR) experiments were performed to examine if the association occurs in a reduced system containing only purified Pin1 and the N-terminus peptides of PSD-95. The synthetic peptides were immobilized in the streptavidin-coated sensor chip *via* a biotin moiety. Only T19 and S25 peptides were used as baits since they are the only known sites implicated in activity-dependent forms of synaptic plasticity (Morabito et al., 2004; Nelson et al., 2013). The immobilized peptides were challenged with purified Pin1 proteins, and the association was scored as an increase in resonance units as a function of time (RU values). Wildtype Pin1, the WW domain of Pin1, and the RR/AA mutant all bound well to the phospho-T19 peptide (only data for wt Pin1 is shown). Only wildtype Pin1 bound both phospho-T19 and phospho-S25 (Figure 2E). No detectable binding was observed to non-phosphorylated peptides or GST/BSA, used as negative controls (data not shown). These results suggest that in cells, the WW domain of Pin1 can bind the phosphorylated residues in the N-terminus domain of PSD-95.

### The α-Chymotrypsin-Coupled *Cis-Trans* Isomerization Assay Reveals Sites of Binding and Isomerization

Phosphorylation of S/T-proline bonds triggers conformational changes in protein structure by transiently changing the cis-trans equilibrium of the peptidyl-prolyl amide bonds (Lu et al., 2007). The peptidyl-prolyl amide bonds can exist in four possible conformations: (1) *cis* non-phosphorylated, (2) *trans*-non-phosphorylated, (3) *cis* phosphorylated, and (4) *trans* phosphorylated. Because Pin1 is the only phosphorylation-dependent peptidyl-prolyl isomerase in most eukaryotic cells, I used the α-chymotrypsin *cis/trans* isomerization assay which is used to follow in real-time the *cis/trans* isomerization of a phosphorylated peptidyl-prolyl bond (Fischer, 1994). This assay takes advantage of the inability of α-chymotrypsin to hydrolyzed peptide bonds when the amino acid preceding the proline is in the *cis* configuration. Therefore, this assay was modified to work with full proteins in lysates and confirm the site of Pin1 binding and isomerization on full-length PSD-95. In addition, I also tested if phosphorylation of T19 and S25 alters the local conformation of the N-terminus domain.

The purified Pin1 protein was catalytically active as evidence of the accelerated loss of the Suc-AEPY-pNA peptide, measured at 390 nm (Figure 3A; Fischer, 1994). On full-length PSD-95, the degradation by α-chymotrypsin was measured *via* Western immunoblotting and the intensity of the band corresponding to full-length PSD-95 was quantified (Figure 3B). Homogenates incubated with wt Pin1 showed a time-dependent loss of full-length PSD-95 intensity when compared to the isomerization mutant (RR/AA; Figure 3C). To further explore the sites of Pin1 association/isomerization, T19, S25, and S35 were mutated to alanine (referred to as N3A, short for three N-terminus residues mutated into alanine; Figure 4A). Mutating these sites to alanine residues should occlude Pin1-mediated isomerization because (1) the alanine-proline prolyl bond tends to be in the *trans* configuration 99% of the time (Fischer, 1994), (2) Pin1 does not bind to non-phosphorylated residues, and (3) the Pin1 isomerase domain cannot isomerize these bonds. In contrast to the results obtained with wt PSD-95, reactions containing the N3A-PSD-95 mutant were insensitive to Pin1 (Figures 4E,G *left bar graph*), while homogenates expressing wt PSD-95 show an accelerated loss of PSD-95 (Figures 4B,D, *left bar graph*). Additional controls on mutants of the hinge domain, T287A and S295A phospho-mutant (*C2A*), were sensitive to Pin1 (Figures 4H,J, *left bar graph*, *n* = 8 **p < 0.01 unpaired *t*-test). Thus, indicating that these sites are not sensitive to the configuration of this assay or that they do not undergo much *cis-trans* isomerization. Although singly phosphorylated S290 could be isomerized by Pin1, it is highly unlikely due to bivalency requirements for Pin1 binding/isomerization (Daum et al., 2007; Zhang et al., 2012; Eichner et al., 2016; Rogals et al., 2016). Furthermore, a mutant containing both sets of mutations (*N3A/C2A*) behaved just like the N3A-PSD-95 mutant (Figures 4K,M, *left bar graph*). In all experiments, equal amounts of PSD-95 protein are present across all paired reactions (BSA vs. Pin1, right bar graphs). The lack of action by Pin1 in lysates expressing the N3A-PSD-95 mutant supports the idea that the phosphorylated N-terminus domain is a site of Pin1 binding and isomerization in PSD-95.

### N-Terminal PSD-95 Phosphorylation Alters Its Conformation

The data obtained using the α-chymotrypsin assay supports the idea that phosphorylation of the N-terminus domain in PSD-95 alters the conformation of PSD-95 and Pin1 restores its conformation. To further support this hypothesis, I compared the rates of α-chymotrypsin degradation from homogenates expressing either wt PSD-95 or the N3A-PSD-95 mutant without Pin1. In agreement with the idea that Pin1 isomerizes the N-terminus domain, reactions containing the N3A-PSD-95 mutant showed faster degradation than reactions containing wt PSD-95 (Figures 4N,O, ***p < 0.001 unpaired *t*-tests). These results support the following ideas: (1) Pin1 interacts with T19 and S25 in PSD-95, (2) N-terminal phosphorylation of PSD-95 alters this section of PSD-95, (3) the alanine mutants of T19 and S25 adopt the correct conformation, and (4) Pin1 can restore its structure.

## Discussion

Previous reports have shown that Pin1 interacts with the hinge domain of PSD-95 (Antonelli et al., 2016), and in this report, I show that Pin1 binds to full-length PSD-95 *via* the phosphorylated T19, S25, and S35. It is common for Pin1 to bind multiple regions within the same target. But how can Pin1 bind these distant sites in PSD-95? In the extended conformation, Pin1 is over 7.3 nm in length, which is potentially long enough to position one of its domains close to the hinge and the other domain close to the N-terminus domain of PSD-95. The interaction between Pin1 and PSD-95 could be further facilitated by conformational changes in PSD-95 (Nakagawa et al., 2004; Jeyifous et al., 2016). Given that palmitoylation of PSD-95 strongly regulates its conformation, the interplay between PSD-95 conformational states and PSD-95 palmitoylation may be an integral mechanism regulating the association. Therefore, further studies will be required to better our understanding of the relationships between PSD-95/Pin1 association and how N-terminus domain phosphorylation/palmitoylation regulates this interaction. In an accompanying article, on this issue, we specifically evaluate the functional significance of this interaction.

Although the biochemical studies show that Pin1 accelerates the loss of full-length PSD-95 in the α-chymotrypsin assay Antonelli et al. (2016) observed that Pin1 protected PSD-95 from subtilisin degradation. The reason for this discrepancy is unknown, but the data presented here differs from theirs in many ways. First, I provide data showing the activity of purified GST-Pin1. Second, I show the averages for the reactions. Third, I show evidence of equal loading among the pair of reactions. Fourth, I show the full gels showing the products of proteolytic degradation as shown by others (Umeki et al., 2016). Last, a mutagenesis strategy was employed to confirm the region of Pin1-mediated binding and isomerization, which confirmed the idea that Pin1 mediate the *cis-trans* isomerization of the N-terminus domain of PSD-95.

The α-chymotrypsin data raises several interesting questions about the multiple conformations adopted by the N-terminus domain of PSD-95 and the biological activity of these conformations in neurons. For example, it would be interesting to quantitatively estimate the fractions of these conformations in cells or solution as has been done for the mGluR5 receptor (Park et al., 2013). Because each S/T-P bond can exist in 4 different mutually exclusive conformations, the phosphorylated N-terminus domain of PSD-95 could adopt up to 64 possible conformations. If the other three hinge domain sites are included then up to 1,296 isomers of PSD-95 could be present within a given PSD. Therefore, it is important to identify which isomers of PSD-95 are important for normal excitatory synaptic function.

## Data Availability Statement

The datasets generated for this study are available on request to the corresponding author.

## Ethics Statement

All procedures were in accordance with guidelines of and approved by the Institutional Animal Care and Use Committee at the University of Chicago, Brandeis University, and University of Bordeaux II.

## Author Contributions

JD conceived the initial idea, performed experiments, generated graphs, analyzed data and wrote the manuscript.

## Conflict of Interest

The author declares that the research was conducted in the absence of any commercial or financial relationships that could be construed as a potential conflict of interest.
